# Mitochondrial peroxidase TPx-2 is not essential in the blood and insect stages of *Plasmodium berghei*

**DOI:** 10.1186/1756-3305-5-252

**Published:** 2012-11-12

**Authors:** Hirono Masuda-Suganuma, Miho Usui, Shinya Fukumoto, Noboru Inoue, Shin-ichiro Kawazu

**Affiliations:** 1 National Research Center for Protozoan Diseases, Obihiro University of Agriculture and Veterinary Medicine, Inada, Obihiro, 080-8555, Japan

**Keywords:** Peroxiredoxin, Plasmodium berghei, Thioredoxin peroxidase

## Abstract

**Background:**

Malaria parasites actively proliferate in the body of their vertebrate and insect hosts, and are subjected to the toxic effects of reactive oxygen species. The antioxidant defenses of malaria parasites are considered to play essential roles in their survival and are thus considered promising targets for intervention. We sought to identify the cellular function of thioredoxin peroxidase-2 (TPx-2), which is expressed in the mitochondria, by disrupting the TPx-2 gene (*pbtpx-2*) of the rodent malaria parasite *Plasmodium berghei*.

**Findings:**

In three independent experiments, two disruptant populations (TPx-2 KO) and three wild-type parasite populations with pyrimethamine resistance (*dhfr-ts/mt* at the DHFR-TS locus) and intact *pbtpx-2* (TPx-2 WT) were obtained and cloned. Null expression of TPx-2 in the KO population was confirmed by RT-PCR and Western blot analyses. The TPx-2 KO parasite developed normally in mouse erythrocytes and multiplied at a rate similar to that of the TPx-2 WT parasite during the experimental period. The peak period of gametocytemia was delayed by 1 day in the TPx-2 KO compared with that of the TPx-2 WT and the parent parasite, however, the highest gametocyte number was comparable. The number of midgut oocysts in the TPx-2 KO at 14 days post feeding was comparable to that of the TPx-2 WT.

**Conclusions:**

The present finding suggests that mitochondrial Prx TPx-2 is not essential for asexual and the insect stage development of the malaria parasite.

## Findings

### Introduction

Malaria is a disease caused by infection with protozoan parasites of the genus *Plasmodium* and transmitted by *Anopheles* mosquitoes. Almost half of the world’s population lives with a serious risk of contacting malaria, and nearly 1 million people died from the disease in 2010 [1]. Despite years of intensive research, an effective vaccine is still not available, and the parasite displays increasing resistance towards the commonly used anti-malarial drugs. To combat malaria, a better understanding of the basic biology of the parasite is needed, especially with regard to the mechanisms of adaptation to environmental conditions.

As *Plasmodium* spp. actively proliferate in the erythrocytes of their vertebrate hosts, large quantities of reactive oxygen species (ROS), which damage biological macromolecules, are generated in the cell during parasite development [2,3]. One of the major sources of ROS in the parasite cell is heme, which is produced as a byproduct of hemoglobin digestion for amino acid procurement [4]. In addition, the parasite possesses a mitochondrion with a functional electron transport chain. During respiration, ROS are generated that need to be removed. ROS also emerge in the cell when the organism is exposed to a variety of stress conditions such as the action of the host immune system [5]. Since *Plasmodium* spp. are highly susceptible to oxidative stresses, their antioxidant defenses are considered to play essential roles in their development, and they are thus expected to be potential targets for chemotherapy [6].

Malaria parasites possess six peroxidases localized in the cytoplasm, mitochondrion, apicoplast and nucleus: a 1-Cys Prx, two typical 2-Cys Prxs, a 1-Cys antioxidant protein (AOP), a Prx family with unusual biochemical characteristics and a glutathione (GSH) peroxidase-like thioredoxin peroxidase (TPx_Gl_) [7-9]. Since malaria parasites do not possess catalase and genuine GSH peroxidase in their genome, it is considered that GSH itself is the major redox buffer for transient H_2_O_2_ exposure and the basal peroxide flux in the cell is dealt with by the Trx system [6]. It has recently been reported that disruption of the gene for the cytosolic 2-Cys Prx (TPx-1) in *Plasmodium* renders parasites hypersensitive to ROS and reactive nitrogen species, although it did not affect parasite growth under normal *in vitro* and *in vivo* asexual development [10]. Thus far, however, the functions of the Prx family in the organelle are unclear. In the present study, the gene coding for TPx-2, which is expressed in the mitochondrion of the rodent malaria parasite *P*. *berghei* (Additional file 1: Figure S1), was disrupted and phenotypes of the disruptant were observed in experimental infections in mice and mosquitoes in order to investigate unidentified cellular function of the Prx family in malaria parasites.

### Methods

The *P*. *berghei* ANKA strain was obtained from the Armed Forces Research Institute of Medical Sciences, Thailand. The parasite was maintained by mosquito transmission in *Anopheles stephensi* interspersed by a maximum of two serial passages in BALB/c mice (Clea Tokyo, Japan).

For disruption of the *pbtpx-2*, 5′ and 3′ portions of the gene were amplified by polymerase chain reaction (PCR) and cloned into the targeting vector pMD204 [11], which was supplied by the Malaria Research and Reference Reagent Resource Center; MR4/ATCC, Manassas, VA, USA. Each fragment contained part of the coding sequence and flanking region and was amplified with sequence-specific primers and parasite genomic DNA. The primers used for the 5^′^ fragment were 5^′^GGG CCC CTA TCT GAG TAA TAT CTC ATA TCT CC-3^′^ and 5^′^GAG CTC CAT GAG AAA ACG ATC TCA CTG ATC-3^′^ (*Apa* I and *Xho* I sites are underlined). The primers used for the 3^′^ fragment were 5^′^GAA TTC GTT TAG ACC CAA TAA TGA GGC-3^′^ and 5^′^CTG CAG GTT TTT CAC ACA CAG GAG TTA C-3^′^ (*Eco* RI and *Pst* I sites are underlined). The primers were designed on the basis of sequences in the *P*. *berghei* genome database provided by the *Plasmodium* Genome Resource [PlasmoDB: PBANKA_143080]. PCR products were purified and cloned into the upstream or downstream of the pyrimethamine-resistant form of the *P*. *berghei* dihydrofolate reductase-thymidylate synthase gene (*dhfr-ts*), which was used as a selectable marker. For gene targeting experiments, the plasmid was digested with *Apa* I and *Pst* I to separate the linear targeting construct from the plasmid backbone.

Transfection was performed according to the protocol of Janse *et al*. [12]. To transfect the knock out construct into *P. berghei* with Nucleofector® (Amaxa, Germany), mature schizonts were prepared. To collect the parasites, ICR mice (Clea Japan) were infected with *P. berghei* by intraperitoneal (i.p.) injection of 5 x 10^6^ parasitized erythrocytes per animal. The schizont pellet was resuspended in 0.1 ml Nucleofector® working solution containing 9 μg linearized targeting vector, then transferred to electroporation cuvettes for transfection at the U-33 setting. After transfection, 50 μl of complete culture medium was added to the mixture and was immediately inoculated into a mouse per transfection by intravenous (i.v.) injection. The mice were provided with drinking water containing pyrimethamine (0.7 mg/ml) 1 day after infection with transfected parasites. When parasitemia had reached 1-2%, the parasite population in each mouse was examined by allele-specific PCR prior to cloning the parasite population with the wild-type and disruptant allele by limiting dilution. Both parasite genomes were determined by PCR and Southern blot analyses. Null-phenotype for TPx-2 expression was confirmed by reverse transcriptase PCR (RT-PCR) and Western blotting.

Five–week-old female BALB/c mice (CLEA Japan, Japan) were infected with *P*. *berghei* (10^6^ parasitized erythrocytes per mouse) by i.p. injection. Parasitemia and gametocytemia were determined by microscopic examination of Giemsa-stained thin blood films. Gametocytes were distinguished by size and coloration. The animal experiments in this study were carried out in compliance with the Guide for Animal Experimentation at Obihiro University of Agriculture and Veterinary Medicine (permission numbers: 21–56 and 22–48).

Six-week-old male BALB/c mice (CLEA Japan, Japan) were infected with *P*. *berghei* by i.p. injection of the parasite, which had been stored as frozen stock at −80°C. Parasitemia of each mouse was monitored daily by light microscopic observation of Giemsa-stained thin blood smears. *A. stephensi* mosquitoes were maintained on 10% sugar solution at 27°C and 80% relative humidity under a 12 hr light/dark cycle. When the number of microgametocytes that could exflagellate *in vitro*[13] had reached 20–30 per 1 x 10^5^ erythrocytes, the mosquitoes were fed on the mice for 2 h at 19°C. The parasite-infected mosquitoes (100–200 mosquitoes per group) were maintained at 19°C with 10% sugar solution.

At 14 days post-feeding, mosquitoes that had been infected with parasite populations were dissected and their midguts were isolated. The midguts were stained using the improved technique [14]. Briefly, the midguts were stained with 0.5% mercurochrome (Sigma Aldrich Japan, Japan) in water at room temperature for 10 min and washed in PBS for 30 min. Next, stained midguts were fixed with 10% formalin (Wako Pure Chemical, Japan) for 24 h and opened along the median line. The midgut specimens were observed, and the numbers of red-stained oocysts were counted using light microscopy (x 100 or x 200).

Differences were evaluated with the Student’s *t*-test (mouse infection) or the Mann–Whitney test (mosquito infection). *P* < 0.05 was considered statistically significant.

### Results and discussion

**Figure 1:**
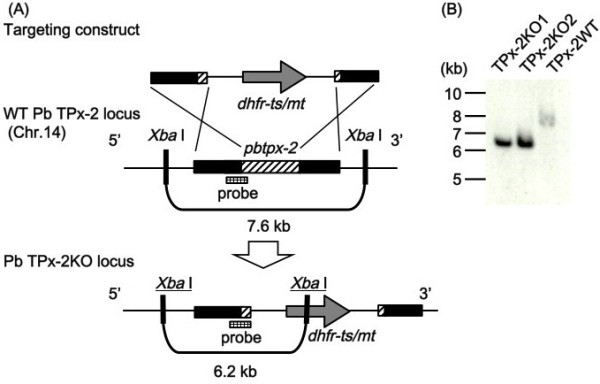
**Targeted disruption of the *tpx-2 *gene in *P. berghei. *** (**A**) The targeting construct, composed of the 5^′^ and 3^′^ regions (filled boxes) and partial open reading frame (hatched boxes) of the *pbtpx-2* and the pyrimethamine-resistant variant of *dhfr-ts* of *P*. *berghei* (*dhfr-ts/mt*) as a selectable marker (gray arrow), was integrated into the wild-type (WT) PbTPx-2 locus by double-crossover homologous recombination. Recombination disrupts *pbtpx-2* and creates the locus containing *dhfr-ts/mt* (PbTPx-2 KO locus), which confers pyrimethamine resistance to disruptants. (**B**) Southern blot analysis of genomic DNA samples from the wild-type parasite populations with pyrimethamine resistance (*dhfr-ts/mt* at the *dhfr-ts* locus) (TPx-2 WT), and two *pbtpx-2* disruptant populations (TPx-2 KO). DNA samples were digested with *Xba* I, separated on 0.7% agarose gels, transferred to nylon membranes, and hybridized with a probe. The single 6.2-kb band in the TPx-2 KO populations indicates *pbtpx-2* disruption, whereas the single 7.6-kb band in the TPx-2 WT populations indicates an intact *pbtpx-2* locus.

**Figure 2:**
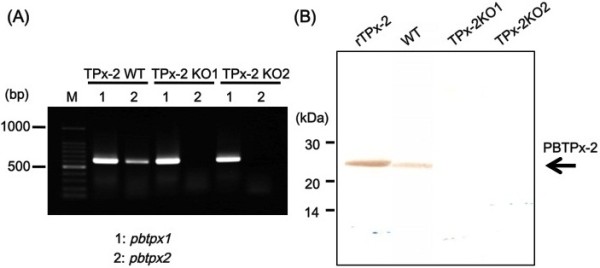
**Verification of the TPx-2 null phenotype in gene-disrupted *P. berghei. *** WT, TPx-2 WT, and TPx-2 KO parasite populations were inoculated into mice, and parasite-infected erythrocytes, total protein, and total RNA of the parasite cells were prepared [15]. (**A**) The absence of TPx-2-specific mRNA expression in TPx-2 KO was examined by RT-PCR analysis. Total RNA samples (1 μg) were reverse transcribed and the cDNAs were amplified with the sequence-specific primer pairs that amplify *pbtpx-1* (PBANKA_130280) and *pbtpx-2* (PBANKA_143080)*.* The primers used were: 5^′^-CGG AAT TCA TGC CAT CAA TTG TAG GAA ATC AAG CC-3^′^ and 5^′^-GCG GAT CCT TAC AAA CTC GAT AAA TAT TTT TGC AAC TCC-3^′^ for *pbtpx-1*, and 5′-GGA TCC TCT CAT GTT ACT CAG AAG GC-3^′^ and 5^′^-TAG CCT CGA GTT ACT TTT TGT ATT C-3^′^ for *pbtpx-2*, respectively. Amplification of *pbtpx-1* but not *pbtpx-2* from TPx-2 KO cDNAs indicates the absence of TPx-2-specific mRNA expression in this population. Molecular size markers in bp are indicated on the left. (**B**) The absence of TPx-2 protein in TPx-2 KO was examined by Western blot analysis [15]. Total protein samples (5 μg) together with recombinant TPx-2 protein (rTPx-2; 5 ng) were separated by sodium dodecyl sulfate (SDS)-polyacrylamide gel electrophoresis (PAGE; 12.5%) and probed with anti-recombinant PbTPx-2 (rPbTPx-2) rabbit serum (1:1000). Anti-rPbTPx-2 rabbit serum was prepared as previously described [16] except that pGEX-6P-1 (Amersham Biosciences) was used. Protein size markers in kDa are indicated on the left.

Merozoites in segmented schizonts were transfected with the targeting construct by nucleofection and were subsequently inoculated into naïve mice. Integration of the construct into the *pbtpx-2* locus by homologous recombination resulted in disruption of the single-copy gene and insertion of a selectable marker, the *dhfr-ts* with a pyrimethamine-resistance mutation (*dhfr-ts/mt*), at this locus (Figure 1A). Parasites with *pbtpx-2* disruption were selected by treatment with pyrimethamine. The allele-specific PCR analysis showed that parasites selected with pyrimethamine were a mixture of wild-type parasites and *pbtpx-2* disruptants (data not shown). Parasite populations were separated into two groups by limiting dilution and subsequent inoculation into 37 rats. In three independent electroporation experiments, two disruptant (TPx-2 KO) and three wild-type parasite clones with pyrimethamine resistance (*dhfr-ts/mt* at the *dhfr-ts* locus) and intact *pbtpx-2* (TPx-2 WT) were established. In the first experiment, three TPx-2 WT clones were obtained. Each TPx-2 KO clone was obtained from the second and third experiments, respectively. Southern blot analysis confirmed the replacement of the *pbtpx-2* locus by the targeting construct in the TPx-2 KO genome (Figure 1B). Null expression of TPx-2 in the KO clone was confirmed by RT-PCR and Western blot analyses (Figure 2). The *dhfr-ts* locus of the TPx-2 WT clone was amplified by PCR and sequenced, and replacement with the pyrimethamine-resistance mutation was confirmed (data not shown).

**Figure 3:**
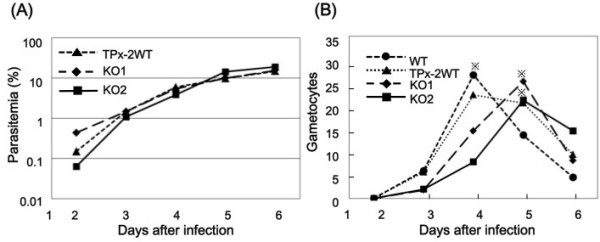
**Infection of mice with *tpx-2 *-disrupted *P. berghei. *** Parasite populations, including WT, TPx-2 WT and two TPx-2 KO populations (KO1 and KO2), were inoculated into mice, and the courses of parasite development in erythrocytes were observed. (**A**) Changes in parasitemia 6 days after infection (three mice per group). (**B**) Changes in gametocytemia 6 days after infection (four mice per group). Gametocyte numbers were shown as gametocytes/10^4^ erythrocytes. Data show mean values of parasitemia percentage (A) and gametocyte number (B). *Significant difference in the days required for having the highest gametocyte number between WT and TPx-2 KO populations (*P* < 0.05; Student’s *t*-test).

*In vivo* asexual growth was compared in TPx-2 KO and TPx-2 WT clones. For these experiments, BALB/c mice were infected with the parasite clones by i.p. injection, and the course of parasitemia was determined. The TPx-2 WT clone showed equal levels of development and multiplication within erythrocytes, similar to the parent strain (WT) (data not shown), and they showed a high level of parasitemia (>5%) 4 days after infection (Figure 3A). The course of parasitemia observed in TPx-2 KO1-infected mice was similar to that of TPx-2 WT-infected mice. This phenotype was confirmed in TPx-2 KO2, which was obtained from an independent electroporation experiment (Figure 3A). There was no difference in the morphology of parasite cells between two TPx-2 KO, TPx-2 WT, and WT clones (data not shown). To evaluate the effect of *pbtpx-2* disruption on development of sexual-stage parasites, the numbers of gametocytes in parasite-infected blood were counted and compared among TPx-2 KO1-2, TPx-2 WT and WT (Figure 3B). In TPx-2 WT-infected mice, gametocytes were observed from 2 days after infection; they increased in number with parasitemia progression and peaked 4 days after infection. The course of gametocytemia observed in TPx-2 WT-infected mice was similar to that of WT-infected mice. Gametocytemia, recorded as the number of gametocytes/10^4^ erythrocytes in TPx-2 WT and WT-infected mice at 4 day of infection, was 19.0 ± 12.0 and 26.0 ± 10.0, respectively (*P* > 0.05). In TPx-2 KO1-2-infected mice, gametocytes were also observed from 2 days after infection but peaked 5 days after infection. The peak of gametocytemia in TPx-2 KO-infected mice was thus delayed by 1 day compared with that of Prx WT- and WT-infected mice. The difference between WT and TPx-2 KO populations in the number of days required for having the peak gametocyte number was significant (*P* < 0.05). Gametocytemia, recorded in TPx-2 KO1-2-infected mice as the number of gametocytes/10^4^ erythrocytes on day 5 of infection, was 28.0 ± 9.0 and 24.0 ± 8.0, respectively. The peak number of gametocytes in TPx-2 KO1-2-infected mice was thus comparable to that of TPx-2 WT-infected mice (*P* > 0.05).

**Figure 4:**
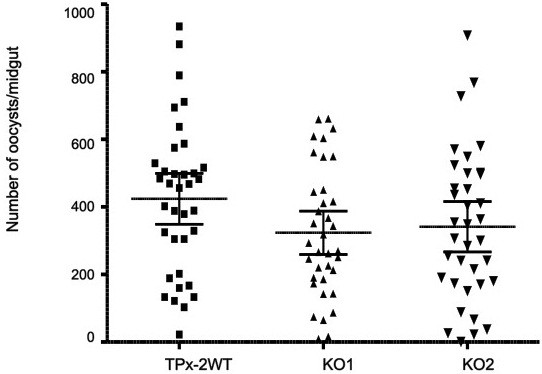
**Number of oocysts per midgut.** Data were obtained from TPx-2 WT and two TPx-2 KO populations (KO1 and KO2). The line indicates the 95% confidence interval of the mean. The oocyst formation rate of the TPx-2 KO populations was the same as for the TPx-2 WT population (*P* > 0.05; Mann–Whitney test). The number of mosquitoes dissected was 36 for each experimental group. The figure shows representative results of three independent experiments.

To investigate the effect of *pbtpx-2* disruption in the insect stage, mosquitoes were fed on parasite infected BALB/c mice that had been infected with TPx-2 WT and two TPx-2 KO populations. At 14 days post-feeding, the mosquitoes were dissected, and oocyst numbers in the midgut were counted. In this experiment, the number of midgut oocysts in TPx-2 KO populations at 14 days post-feeding was comparable to those of the TPx-2 WT (*P* > 0.05) (Figure 4). There was no significant difference in the developmental stages of oocysts between TPx-2 KO and TPx-2 WT populations (data not shown), and this finding suggested that TPx-2 KO oocysts could develop similarly to those of TPx-2 WT in the midgut.

The results presented here suggest that *pbtpx-2* disruption does not affect asexual intraerythrocytic growth of parasites. The present findings might be contradicted by the facts that the mitochondria of the rodent malaria parasite, *P. yoelii*, possess an active respiratory chain [17] and the mitochondrial peroxide-detoxifying capacity is likely to be provided by Prx [18]. It has also been reported in other organisms that a mitochondrial Trx system plays pivotal roles in defense against oxidative stress generated in the organelle [19].

In *P. falciparum*, it had been believed for a long time that the parasite electron transport chain was not a source of ATP, but was essential in the parasite for maintenance of mitochondrial membrane potential, as indicated by their hypersensitivity to proguanil, a drug that collapsed the membrane potential in the presence of an electron transport inhibitor [20,21]. However, it has been shown recently that metabolism in *P. falciparum* grown in human patients is affected by varied oxygen and substrate levels and by host-parasite interactions. As a consequence, the parasite seems to induce expression of genes associated with oxidative phosphorylation as a response against starvation status [22]. The parasite may require TPx-2 for development under such stressful conditions. The phenotype of TPx-2 KO during asexual growth under low glucose supply would be an interesting subject of investigation.

*Plasmodium* mitochondria are morphologically different between the sexual and asexual blood stages [23]. Asexual stage parasites have a tubular-like cristate mitochondrion. In contrast, the mature gametocyte stage has multiple (4–8) mitochondria with greater numbers of cristate structures. The differences suggest that the gametocytes might have a higher demand for energy transduction and are also more metabolically active than the asexual stages [23]. Although the contribution of TPx-2 in gametocyte development remains unknown, the phenotype of TPx-2 KO in the gametocyte stage is thus an interesting subject for further investigation.

The results from phenotype observation in the insect stage suggest that *pbtpx-2* disruption does not affect gamete fertilization, ookinete formation and transformation of the ookinetes to oocysts, which requires ookinete invasion of epithelial cells and attachment to the basal lamina of the mosquito’s midgut. However, it has been reported that ookinetes are subject to oxidative stress during their invasion of midgut epithelial cells [24]. In addition, it is postulated that ROS is produced in the motile ookinete stage, when oxygen metabolism in the cell may be elevated. The expression profiles of the molecules of the Prx family in the TPx-2 KO ookinete would also be worth investigating.

### Conclusions

The present finding suggests that mitochondrial Prx TPx-2 is not essential for the asexual and insect developmental stages of the malaria parasite. Under more pronounced stress conditions, specific functions of TPx-2 may become evident. An explanation for the lack of phenotype in the TPx-2 KO parasites in the blood and insect-stages might be due to redundancy in the function of the multiple members of the thioredoxin system [13]. A multiple Prx KO clone has not yet been produced in *P. berghei.* It will be interesting to develop such KO clones in the future to clarify the Prx family, which may functionally overlap with TPx-2 in the malaria parasite.

## Abbreviations

GSH: Glutathione; Prx: Peroxiredoxin; TPx: Thioredoxin peroxidase; ROS: Reactive oxygen species.

## Competing interests

The authors have no competing interests.

## Authors’ contributions

HMS and MU: designed and performed the experiments and redacted the manuscript. SF, NI and SIK: contributed to the study design and edited the manuscript. All authors read and approved the final manuscript.

## Supplementary Material

Additional file 1 **Figure S1.** Alignment of the deduced amino acid sequences of Plasmodium berghei TPx-2 (PbTPx-2), with those of TPx-2 from other Plasmodium species. Click here for file
