# Supplementary figures and images for: Mitochondrial peroxidase TPx-2 is not essential in the blood and insect stages of Plasmodium berghei
Source: Parasit Vectors. 2012 Nov 12;5:252. doi: 10.1186/1756-3305-5-252 (PMC3507878; doi:10.1186/1756-3305-5-252)

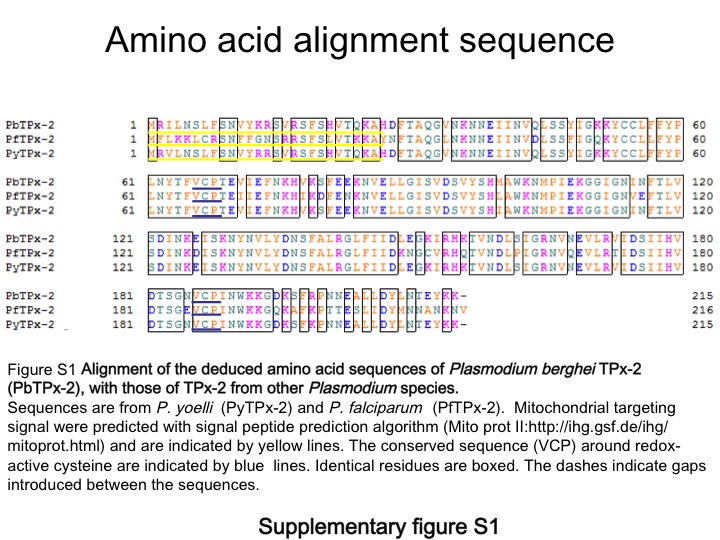

Supplement: Additional file 1 — Figure S1. Alignment of the deduced amino acid sequences of Plasmodium berghei TPx-2 (PbTPx-2), with those of TPx-2 from other Plasmodium species. [file 1756-3305-5-252-S1.jpeg]
